# Hepatocellular Carcinoma Masquerading as Heart Failure: When POCUS “Incidentally” Detects Cancer

**DOI:** 10.24908/pocusj.v11i01.19738

**Published:** 2026-04-22

**Authors:** Luke Shenton, Wesley Chow, Bruce Kimura

**Affiliations:** Departments of Graduate Medical Education and Cardiology, Scripps Mercy Hospital, San Diego, CA, USA

**Keywords:** Hepatocellular Carcinoma, Dyspnea, Inferior Vena Cava Occlusion, Pulmonary Embolism, Cardiac Limited Ultrasound Exam, CLUE, Point of Care Ultrasound, POCUS

## Abstract

Hepatocellular carcinoma (HCC) is a leading cause of cancer-related death worldwide, and cardiovascular involvement can occur in advanced cases. An 84-year-old man with subacute dyspnea on exertion and pedal edema was referred to the cardiologist for suspected heart failure. Point of care ultrasound (POCUS) revealed scant ascites, a liver mass, and inferior vena cava (IVC) tumor thrombus extending into the right atrium. Computed tomography confirmed HCC with extensive intravascular spread and pulmonary emboli. This case illustrates a rare phenomenon of HCC masquerading as heart failure and underscores the potential value and challenges of incorporating POCUS into outpatient evaluations for earlier cancer detection.

## Introduction

Hepatocellular carcinoma (HCC) poses a major global health burden, with over 850,000 new cases and 750,000 deaths in 2022 alone [1]. Prognosis remains poor, with a five-year survival rate of only 18% [2]. Aggressive tumor biology often leads to vascular invasion: tumor thrombi in hepatic veins and the inferior vena cava (IVC) are common in advanced cases [3]. While cirrhosis is a known risk factor, and biannual surveillance with abdominal ultrasound is recommended in patients with cirrhosis, this screening method is imperfect [4]. Notably, no data support HCC detection via routine in-office examination.

Point of care ultrasound (POCUS) is increasingly utilized across specialties. In internal medicine, focused ultrasonography protocols, such as the cardiac limited ultrasound exam (CLUE), can augment bedside assessment [5]. POCUS improves diagnostic sensitivity by revealing findings that elude traditional examination [5,6]. A growing body of literature shows POCUS can aid in the early detection of occult malignancies during outpatient or emergency evaluations. Reports include tumor findings in the colon, stomach, head and neck, breast, kidney, ovary, and even heart—prompting timely workups [7,8]. Here, we report a patient referred for mild dyspnea, who was found to have extensive HCC on an initial cardiac POCUS exam.

## Case presentation

**Figure 1.**
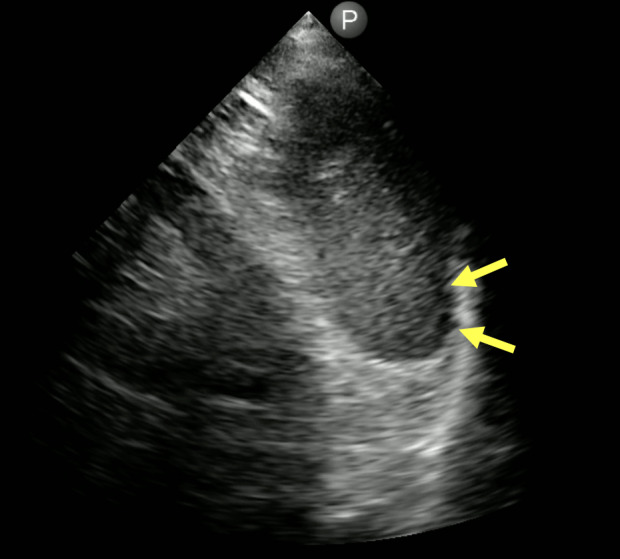
Mild ascites present when imaging the left lower thorax. A small amount of fluid is seen between the diaphragm and spleen (yellow arrows). [Philips Lumify 3.5 MHz cardiac transducer; right (cephalad direction)].

**Figure 2.**
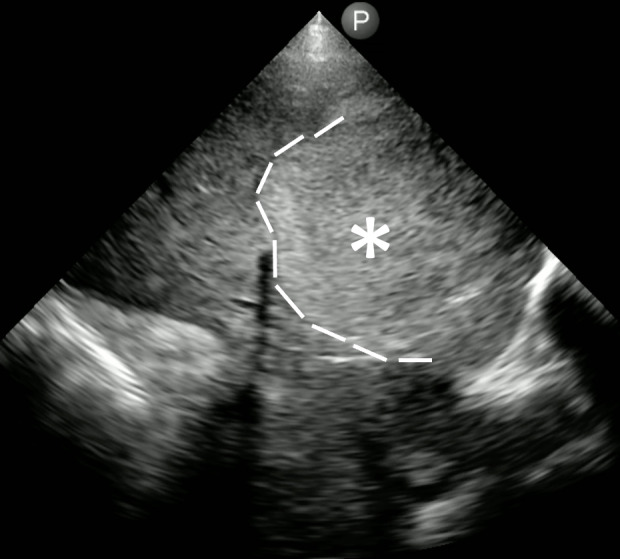
Evidence of a hyperechoic mass (*) in the liver, with notable absence of hepatic vessels. [Philips Lumify 3.5 MHz cardiac transducer; right (cephalad direction)].

**Figure 3.**
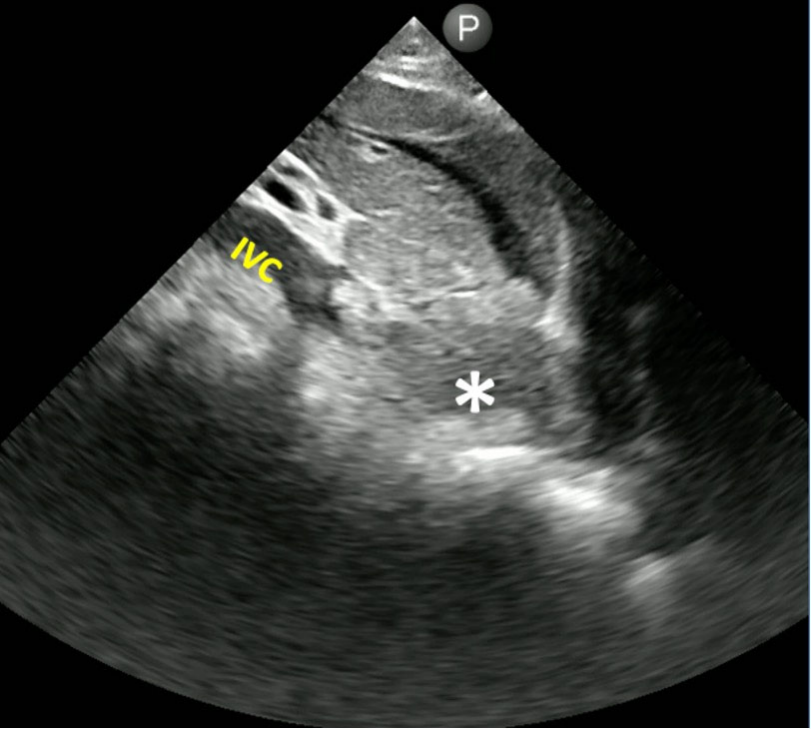
Inferior vena cave (IVC) appears occluded in the proximal portion by an infiltrating mass. [Philips Lumify 3.5 MHz cardiac transducer; right (cephalad direction)].

An 84-year-old man with exercise-induced heart block, sick sinus syndrome requiring a dual-chamber pacemaker, and Parkinson's disease with dysautonomia presented to cardiology on referral from his primary care provider for mild dyspnea on exertion and pedal edema. He had been in his usual health and walking daily, but over several months, developed progressive dyspnea on exertion which limited him to 100 feet. He denied paroxysmal nocturnal dyspnea, orthopnea, pleuritic chest pain, cough, or fever. His routine labs (metabolic panel, blood counts) from a month prior were normal. He had no smoking or drug use history and drank one cocktail nightly. Vital signs and physical exam, including the jugular veins, were unremarkable, aside from 1+ ankle pitting edema bilaterally. As part of the bedside physical examination, a POCUS protocol called CLUE was performed using a standardized six-view protocol (parasternal long-axis, bilateral lung apices, bilateral lung bases, and subcostal/IVC views) to evaluate for left ventricular systolic dysfunction, left atrial enlargement, apical lung B-lines, pleural effusions, and elevated central venous pressure [5]. The CLUE revealed incidental scant ascites instead of pleural effusion on imaging the left lower thorax (Figure 1), and a hyperechoic hepatic mass with absence of hepatic vessels (Figure 2). Imaging of the IVC demonstrated proximal obliteration by an infiltrating mass (Figure 3).

**Figure 4.**
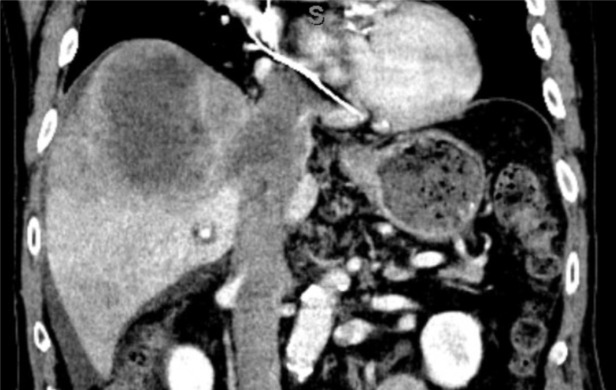
Chest/abdomen computed tomography (CT) with contrast (coronal view) shows a mass in the liver dome with extensive vascular invasion.

The incidental findings and immediate concern for issues beyond heart failure were discussed with the patient and his family. Instead of a trial of empiric diuretic therapy or referral for echocardiography, the patient was urgently referred for a same-day computed tomography (CT) scan. Imaging revealed a 9 × 8 × 10 cm mass in the haptic dome with vascular invasion, including occlusion of the right portal vein and the right and middle hepatic veins, near occlusion of the left hepatic vein, and tumor thrombus extending into the IVC and right atrium (Figure 4). Acute pulmonary emboli were also seen in the left lower, middle, and right lower lobes. At the time of hospital admission, the alpha-fetoprotein level was elevated at 1,503 (Ref Range: 2 - 8.78 ng/mL). Magnetic resonance imaging (MRI) confirmed necrotic hepatocellular carcinoma with thrombus extending into the hepatic veins, IVC, and right atrium. The tumor was deemed unresectable, and palliative immunotherapy was considered. However, he developed worsening dyspnea and weight gain with progressive renal/liver failure. Due to clinical decline, he transitioned to comfort care and passed away.

## Discussion

This case highlights HCC discovered in an elderly patient referred to cardiology for presumed heart failure, who was ultimately found to have malignancy with extensive intravascular tumor spread. HCC often presents late due to nonspecific or absent early symptoms [2]. Cardiac involvement—via obstruction of venous return or inflow—is rare but can manifest as edema, fatigue, and exertional dyspnea. When HCC is suspected, diagnostic imaging is usually contrast-enhanced CT or MRI [9].^.^ To our knowledge, this is the first report of initial HCC detection by a POCUS exam and has implications for the value of POCUS in screening for cancer and the scope of POCUS-assisted physical examination.

This case exemplifies the benefit of outpatient practitioners incorporating POCUS into their in-office exams to expedite diagnosis [5]. POCUS-assisted physical examination can improve the centuries-old techniques that often lie at the foundation of current disease detection, referral, and healthcare delivery pathways [10]. In terms of certain cancers, such as HCC in patients with cirrhosis, screening can reduce mortality [11]. There is also growing evidence to suggest that POCUS, when used judiciously, can expedite the diagnosis of various cancers in patients with vague or non-specific complaints when compared to conventional pathways, offering a safe, inexpensive adjunct [7,8,12]. Standardized scanning protocols tailored for oncology have been proposed, such as the Focused Assessment with Sonography in Cancer (FASC) exam, which outlines a six-view POCUS survey for finding fluid in patients with cancer [13]. Future studies are needed to validate whether such protocols can improve patient outcomes when implemented in primary care or survivorship clinics. Technological advances may also enhance POCUS utility. For instance, artificial intelligence (AI) algorithms could assist less experienced operators in identifying abnormal POCUS findings or even perform preliminary scans for common pathology. Although no survival benefit was realized in this case, POCUS enabled an expedited outpatient diagnosis of a deadly disease. This allowed for earlier treatment of the patient's pulmonary emboli which potentially slowed clinical deterioration and provided more lead time for the patient and family to discuss plans and arrange affairs.

This case reinforces that exertional dyspnea and pedal edema are not always due to congestive heart failure. In this case, the patient's presentation could be explained by the ominous extracardiac results of an unexpected tumor occluding the IVC and embolizing to the lung. CLUE was performed to evaluate for suspected chronic heart failure, but serendipitously found HCC, technically as an incidental finding. The diagnosis of HCC was not within the scope of the exam, clinical context, or purview of the cardiologist, and its discovery brings to light the medical, legal, and economic complexities of incorporating POCUS into office examination. Accordingly, we do not advocate indiscriminate “head-to-toe” screening with POCUS in asymptomatic patients. Rather, this case supports a cautious, context-driven approach in which focused protocols are anchored to a clear clinical question, and sonographers remain mindful of the downstream implications of indeterminate or incidental findings. Contextual imaging emphasizes POCUS imaging decisions made within specific clinical scenarios or contexts, considering urgency, setting, available equipment, and clinician expertise [14]. This targeted approach, similar to the physical examination, focuses on clinically relevant findings, thus reducing the volume of training or testing that would be necessary to diagnose all incidental or incompletely characterized abnormalities. By streamlining clinical workflows and minimizing documentation burdens, contextual imaging facilitates a broader adoption of POCUS by general practitioners. However, the use of POCUS in the context for oncology screening or general practice comes with challenges. Many clinicians lack POCUS experience, and training programs are still evolving to address this need [15]. Misinterpretation of images could lead to false reassurance or unnecessary alarm. Indeed, widespread use of POCUS in other contexts may increase the incidence of incidental or indeterminate findings that necessitate additional testing. At the current time, clear guidelines on managing incidental POCUS findings are lacking, and clinicians must use judgment on when further evaluation is warranted.

We report a case of an ominous masquerader of chronic heart failure, which was incidentally found by using cardiac POCUS. Ultimately, broader adoption of POCUS may require interdisciplinary consensus on best practices and further outcome-based research to guide its use. Future studies should explicitly incorporate patient-centered outcomes and health-economic endpoints to determine when POCUS-enabled cancer detection improves value, rather than unnecessarily increasing downstream testing and anxiety. Once these challenges are met, a modernized, POCUS-assisted in-office exam could improve the timeliness of cancer diagnosis and care.
